# Compression therapies for the treatment of venous leg ulcers: a mixed method process evaluation in a randomised controlled trial, VenUS6

**DOI:** 10.1186/s13063-026-09854-6

**Published:** 2026-07-02

**Authors:** Maartje Kletter, Jane Griffiths, Catherine Arundel, Jo Dumville, VenUS6 Collaborators, Una Adderley, Ian Chetter, Nicky Cullum, Tom Davill, Catherine Hewitt, Charlotte Hirst, Katherine Jones, Julie Mullings, Charlie Peck, Gareth Roberts, Pedro Saramago, Brigid Smart, Marta Soares, Philip Stather, Luke Strachan, Nikki Stubbs, David Torgerson, Jude Watson, Charlie Welch, Sabeen Zahra

**Affiliations:** 1 Division of Nursing, Midwifery and Social Work, School of Health Sciences, Jean McFarlane Building, University of Manchester, Oxford Road, Manchester, M13 9PL UK; 2 York Trials Unit, Department of Health Sciences, Faculty of Science, University of York, York, YO10 5DD UK

## Abstract

**Background:**

Strong compression treatment is an evidence-based first-line treatment that helps reduce the healing time of venous leg ulcers. VenUS 6 is a three-arm RCT conducted in the UK that aimed to compare 4-layer bandages and 2-layer hosiery with compression wraps and 2-layer bandages. The study found that compression wraps are unlikely to reduce time to venous leg ulcer healing compared to the other treatments evaluate, although there was some uncertainty with this estimate. We nested a mixed-methods process evaluation within VenUS 6 to gain a more in-depth understanding of trial participant and nursing staff views on and experiences of various compression therapies, and to consider how these insights may explain VenUS 6 outcomes.

**Methods:**

We conducted individual, in-depth interviews with 11 trial participants and focus group discussions with 19 relevant health professionals based at seven participating primary, secondary and community care NHS Trust sites. We also used data collected during the trial from patients about their experience of compression use. We conducted a separate UK-wide survey with nurses to learn more about availability of the trial compression treatment options in clinics and their views on the ease of use of the compression treatments in scope. Qualitative data were analysed using framework analysis and quantitative data were analysed descriptively. Data were integrated with the help of the Pillar Integration Process.

**Results:**

There was high satisfaction with compression wraps and limited discomfort among trial participants, who appreciated the wraps could be adjusted and taken off to allow them to take showers. Staff mentioned, however, that trial participants were prone to loosen the wraps. Whilst 2-layer hosiery allowed for self-application and could be taken off, trial participants said the hosiery was very tight and difficult to apply. Bandages, on the other hand, whilst bulky and sometimes uncomfortable, would often not be removed by trial participants, leading to greater exposure to effective compression.

**Conclusion:**

We have identified that people with venous ulcers wearing compression wraps are more likely to report loosening them and removal at night, than people wearing other types of compression treatment which was confirmed by staff. These reported behaviours could explain longer healing times for compression wraps, compared to other treatments delivered in our RCT. Despite longer times to healing, compression wraps may still be an appropriate compression treatment option for some people, and it is important for nursing staff to have conversations with service users and share decisions about suitable treatment options.

## Introduction

Venous leg ulcers (VLUs) are open wounds on the lower leg, caused by damaged or diseased veins that impair blood flow towards the heart. They are common chronic wounds that significantly affect individuals’ health-related quality of life [1–5].

Strong compression treatment (40 mmHg pressure at the ankle) is an evidence-based first-line treatment that helps reduce the healing time of VLUs [6, 7]. This complex intervention includes a range of compression therapies, commonly two- or four-layer compression bandages with an elastic layer, short-stretch bandages (SSB), two-layer hosiery (2LH) and compression wraps (CW), in addition to primary contact dressings applied beneath the compression, and topical treatments, applied as necessary. Compression therapies apply graduated pressure from the ankle to the knee to improve blood flow, but they vary in their mode of application.

Whilst compression bandages normally require application by a trained professional, 2LH and CWs can allow for self-application by patients or their informal carers. Hosiery can however be challenging for people to pull on and off because the stockings need to be tight to apply the required level of pressure, although application aids are available [8]. The CWs use hook and loop fastening and are wrapped around the leg and then tightened, rather than being pulled on and off.

Investigating the relative effectiveness of different compression systems for treating people with active VLUs, a comprehensive individual patient data meta-analyses (*n* = 797) found the four-layer bandage (4LB) to be more clinically effective than the SSB [9]. However, a randomised controlled trial (RCT) of 2LH and the 4LB for treating VLUs (VenUS IV; *n* = 457), found no clear difference in time to healing between the groups [10]. Two-layer compression hosiery was, however, the more cost-effective treatment but this treatment is not suitable for all patients. A wider network meta-analysis and decision analytical model, which included VenUS IV data, suggested that of all compression therapies the two-layer compression bandage (2LB) may be the most clinically and cost-effective treatment, but evidence was highly uncertain [11]. There was also very limited evidence on the clinical and cost effectiveness of CWs as a VLU treatment (two RCTs, *n* = 78 participants) [12, 13], despite their increasing use in routine practice in the UK.

The data presented in this paper are from VenUS 6, a three-arm RCT that compared the relative effectiveness of three different approaches to compression for VLU treatment. The study compared CWs with evidence-based compression (a choice of 4LB or 2LH) and 2LB [14]. VenUS 6 findings suggest that time to healing may be similar for 4LB, 2LH and 2LB but that CW results in a longer time to ulcer healing [15]. Wider assessment of cost effectiveness suggests that CWs are unlikely to be cost effective compared to alternative types of compression (Phung H, Soares MO, Arundel CE, Dumville JC, Saramago P, on behalf of the VenUS 6 investigators: Compression Systems for Venous Leg Ulcers: a Network Meta-Analysis and Cost-Effectiveness Analysis, unpublished).

Patient adherence to compression treatment is notoriously challenging as people can find compression treatments tight, uncomfortable and bulky once applied, which may impact on adherence to the treatment and relative effectiveness [8, 16]. Understanding factors like comfort, pain and wearability are therefore key to ensuring adequate compliance with treatment to promote healing. Furthermore, where compression can be self-applied (e.g. CW or 2LH) there may be differences in patient experience of using these treatments based on mobility, independence and confidence.

As recommended by the UK Medical Research Council (MRC) [17], we therefore nested a mixed-methods process evaluation within VenUS 6. This process evaluation aimed to gain a more in-depth understanding of trial participant and nursing staff views and experiences of various compression therapies, and to consider how these insights may explain VenUS 6 outcomes. Additionally, the process evaluation aimed to learn more about trial processes that can inform implementation of various compression treatments in clinical practice.

## Methods

### Conceptual framework: logic model

**Fig. 1:**
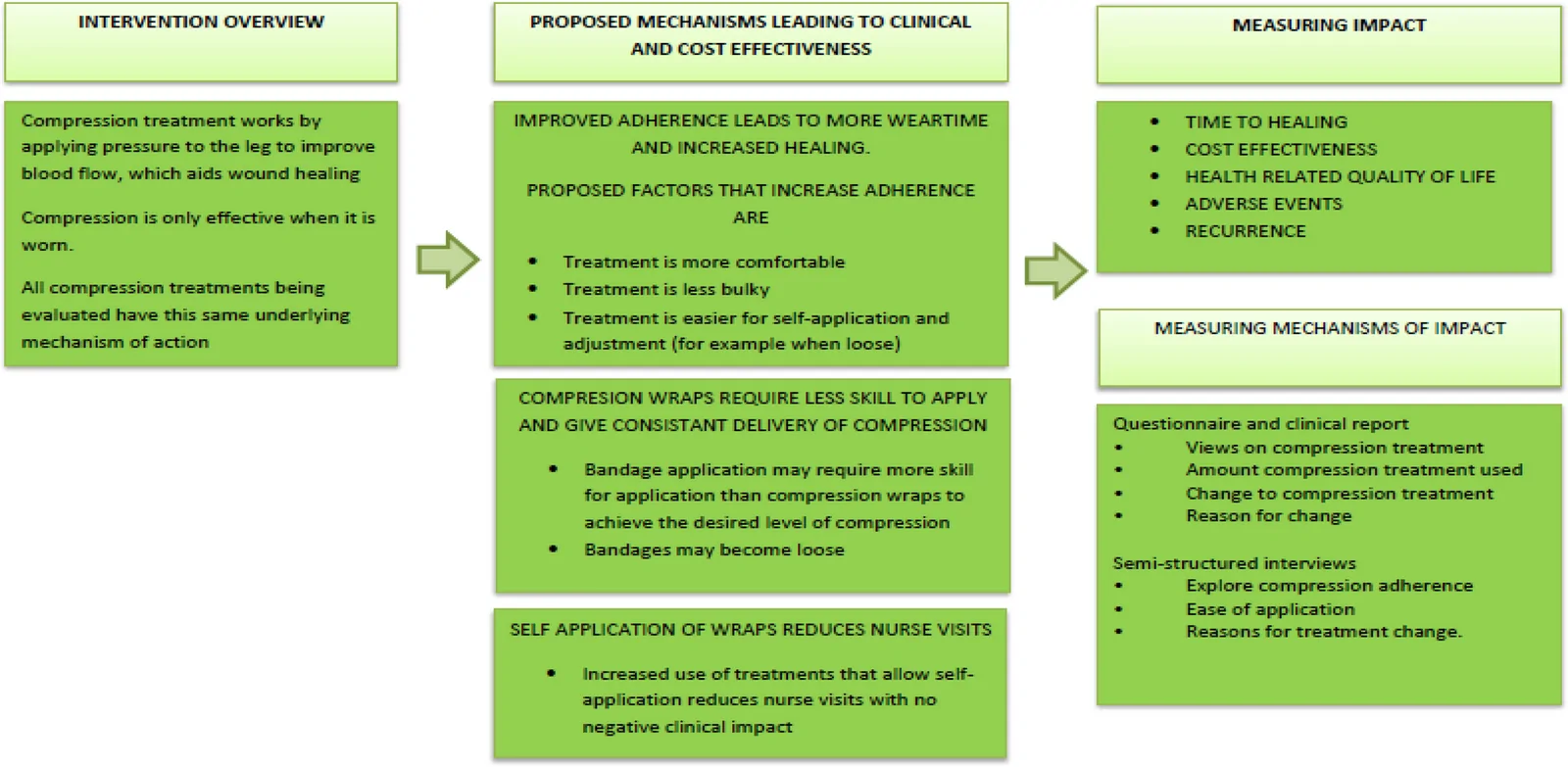
Logic model for conceptualisation of mechanisms of treatment on outcome

As shown in Fig. 1, we developed a logic model to conceptualise the potential mechanisms of treatment on the outcome. It comprised the intervention—compression therapy applied and worn effectively; mechanisms—increased adherence through more comfortable treatments, and compression wraps that give constant compression and can be self-applied; and impact—time to healing.

The protocol for VenUS 6 has previously been published [14] as has a protocol for the process evaluation component [18]. Briefly, we conducted individual in-depth interviews with trial participants, and focus group discussions (FGDs) with relevant health professionals. We also used questionnaires to capture participants’ experience of their compression treatment as part of this process evaluation. We conducted a separate UK wide survey with nurses to learn more about availability of the trial compression treatment options in clinics and their views on the ease of use of the compression treatments included in VenUS 6.

### Trial participant sampling and recruitment

The VenUS 6 consent process included opt-in to be approached about participation in process evaluation interviews. We approached consenting, potential interviewees using a purposive sampling process with the aim of including a maximum variation sample on age, gender and wound-related pain experienced. We oversampled trial participants randomised to CW, because less evidence was available on the implementation of and experiences with CW than for the other trial compression treatments [8].

Recruitment continued until data saturation had been reached, with data reviewed continuously to determine when this was achieved. When no further information was identified in the final few interviews, we stopped interviewing [19].

### Health professional (nurse) sampling and recruitment

We identified, consented, and then recruited nurses involved in providing compression treatment in the VenUS 6 trial at participating sites. Sites were selected based on purposive sampling to achieve as much variation as possible in the type of site (community, primary or hospital sites). We also selected sites according to VenUS 6 trial recruitment rates. During FGDs we explored the context of providing compression treatment during the trial, as well as trial processes, with a particular focus on staff views relating to patient recruitment. The number of staff members present was decided by the site itself to allow for anyone involved in trial implementation and recruitment to be present. Again, we recruited until we believed we had reached data saturation, based on continuous review of data and nothing new coming up.

### Data collection and analyses

Interviews were primarily held via telephone and videoconference (Microsoft Teams) and FGDs by videoconferencing only. Full topic guides are included as supplementary materials. Staff group FGDs included MK, a member of the trial coordination team based at York Trial Unit (YTU) and up to five staff members.

Further patient data for this process evaluation were collected using self-reported questionnaires that were sent to all VenUS 6 participants at 1 month following trial recruitment. The self-reported questionnaire gathered a range of information related to experience of compression use including wound-related pain, treatment application, adherence, satisfaction, discomfort, and ease of use.

Due to challenges in interviewing nurses participating in VenUS 6, we also conducted an anonymous on-line survey (Qualtrics) of UK nurses involved in delivering VLU care, to capture additional insights on how easy staff found it to apply the various compression therapies included in VenUS 6, how staff accessed these therapies and, if ordered specifically for a patient, how long it took for the compression to arrive. Additionally, the questionnaire asked staff how easy they thought patients normally found applying CW or 2LH. The survey was provided to staff at the Society of Tissue Viability conference (2023) and was also distributed via X (formerly Twitter) by the Society of Tissue Viability, the Wounds Research Network and by the VenUS 6 team.

We completed analysis of collected data (interviews with patients, FGDs with staff and survey data) before the trial outcomes were known. Qualitative data were analysed guided by the Framework Method for analysis of qualitative data, as developed by Ritchie and Spencer and applied to healthcare research by Gale et al. [20, 21]. Quantitative data from surveys were analysed using descriptive statistics.

Qualitative process evaluation data were compared with quantitative trial data with the help of the Pillar Integration Process [22]. The first stage was listing. Five columns were developed in Microsoft Excel, two columns on the left for quantitative findings and quantitative categories (grouped data), a central column for the ‘pillar’ and two columns on the right for qualitative findings, including a column for themes and a column including quotes that illustrated the themes. The qualitative data were then matched to the listed quantitative data reflecting patterns, parallels, similarities or differences with the quantitative findings. Once all data were matched, the developed matrix was checked for consistency, and finally the pillar was built. Memos, describing inferences about patterns, insights and themes that emerged were developed and summarised into the ‘PILLAR’ column, which contained the integrated findings from each row.

## Results

### Overview of process evaluation participants

Eleven patients were interviewed. They were aged between 51 and 88 years old at the time of screening for trial participation, and six were female. Six had been assigned to CWs, whilst two and three were assigned to the EBC arm and 2LB arm, respectively (Table 1). Most patients had experienced several of the trial compression treatments either previously, or after healing of the ulcer (when they had been assigned compression for prevention) and were therefore also able to comment on these other treatments. We also report data from 427 trial participants who returned a 1-month questionnaire.

**Table 1:** Overview of interviewed trial participants

Participant	Gender	Age	Assigned to
1	M	73	CW
2	M	51	CW
3	M	61	EBC
4	F	83	CW
5	M	82	2LB
6	F	88	CW
7	F	77	CW
8	F	87	CW
9	M	65	2LB
10	F	72	EBC
11	F	81	2LB

Nineteen staff from seven different sites participated in the FGDs. All staff had been involved in implementation of VenUS 6 and/or recruitment for the trial at their site.

We received 44 completed surveys from UK nurses working with compression therapies.

### Trial processes

Trial participants were positive about trial participation, but due to the time between recruitment and their interview, they were unable to recall details about trial recruitment processes.

A lady came to see me; I don’t know who it was, but she especially came to ask me if I would take part in the trial.—Patient 6, Female

Yes, in fact the very first day I went to the doppler clinic I was asked by the nurse there and then. She said I seemed a perfect candidate and would I agree. To which I obviously said yes.—Patient 10, Female

Staff mentioned that they had expected trial recruitment to be easier than it was. Whilst the trial eligibility criteria were intentionally broad, staff felt that trial enrolment could risk some trial participants being allocated to compression therapies that had previously used but did not like. Staff were concerned this would impact patient experience and cause rapid switching from the allocated compression treatment. In addition, staff noted that when assessing eligibility via case notes, patients had seemed eligible, but by the time they were screened, their ulcers had sometimes healed. Finally, staff mentioned that referrals of new patients to the leg ulcer clinic were not always sufficiently clear to deduce if a patient would potentially be eligible. This was particularly difficult for sites that covered large geographical areas, as travelling to complete eligibility assessment was time consuming and was particularly disheartening if the potential participant was ineligible.

But I think we probably need more recruits. It’s a slow recruitment really and the fact that when patients…Once they started on compression they heal quite quickly, so they are not sometimes then suitable for the study.:—Staff, Site 1

[…] going to the next sort of local clinic to us which is about an hour’s drive away. And again, like the girls said, it’s, you don’t always know what type of… You’ve got the referral in front of you, but what you see in front of you is not necessarily what you get through the door. So, I think that one day I went down to [PLACE] with [COLLEAGUE] and we had four patients through the door and not one of them was eligible. Which was a bit disappointing because it was a decent way to go but it was worth a try.—Staff, Site 3

### Trial participants’ experiences with compression treatments

Overall, across all groups, trial participants mostly reported being satisfied with their allocated compression treatment (Table 2). Trial participants did report that compression bandages could make their legs feel wide, clumsy, and less mobile and that bandage slippage was an issue. We could not distinguish between 4 and 2LB in these comments, mostly because trial participants themselves could not recount the exact number of layers they received. This was due to the fact that whilst we knew what participants were prescribed as per the trial, patients often received different treatments whist they were waiting. It was also sometimes the case that participants did not have any previous experience with bandages, making it difficult for them to comment on the difference between two and four layers.

They did their job they did the trick; they were working ok but I found that they were they didn’t stay up so they turned out to be very uncomfortable, so you know. Sometimes I had to ring and say I’ve got to come in because they slipped down. One time they slipped down so far I had to cut them just to get circulation back in the toes. - Patient 9, 2LB, Female

It was very painful until we set on the four-layer compression bandages that eased the pain in it. It was generally at night.—Patient 10 EBC, Female

**Table 2:** Trial participants reported experience and use of the allocated compression treatment at 1-month post randomisation

	**EBC** (*N* = 153)	**2LB** (*N* = 138)	**CW** (*N* = 136)	**Total** (*N* = 427)
**Satisfaction with allocated compression treatment—*n* (%)**				
Very satisfied	64 (41.8)	80 (58.0)	78 (57.4)	222 (52.0)
Satisfied	72 (47.1)	52 (37.7)	47 (34.6)	171 (40.0)
No view	9 (5.9)	4 (2.9)	5 (3.7)	18 (4.2)
Unsatisfied	5 (3.3)	1 (0.7)	5 (3.7)	11 (2.6)
Very unsatisfied	2 (1.3)	1 (0.7)	1 (0.7)	4 (0.9)
Missing	1 (0.7)	0 (0.0)	0 (0.0)	1 (0.2)
Discomfort experienced when using allocated compression treatment (0–100*)				
N	151	133	131	415
Mean (SD)	30.5 (27.6)	22.7 (21.2)	18.7 (20.8)	24.3 (24.1)
Median (Q1, Q3)	21.0 (6.0, 50.0)	15.0 (5.0, 33.0)	10.0 (2.0, 30.0)	18.0 (5.0, 40.0)
Min, Max	0.0, 98.0	0.0, 80.0	0.0, 90.0	0.0, 98.0
**Patient removal of allocated compression treatment—*n* (%)**				
Yes—whenever I want/wanted to	7 (4.6)	5 (3.6)	23 (16.9)	35 (8.2)
Yes—at certain times	37 (24.2)	21 (15.2)	42 (30.9)	100 (23.4)
Yes—only when they are/were painful	15 (9.8)	9 (6.5)	15 (11.0)	39 (9.1)
No—only my nurse/doctor removes my compression treatment	90 (58.8)	99 (71.7)	50 (36.8)	239 (56.0)
Missing	4 (2.6)	4 (2.9)	6 (4.4)	14 (3.3)
**Perceived effect of allocated compression treatment on pain, *n* (%)**				
Increases ulcer-related pain	6 (3.9)	9 (6.5)	0 (0.0)	15 (3.5)
Causes pain that wouldn’t otherwise be present	15 (9.8)	2 (1.4)	6 (4.4)	23 (5.4)
Decreases ulcer related pain	50 (32.7)	48 (34.8)	55 (40.4)	153 (35.8)
Makes no difference to ulcer related pain	60 (39.2)	57 (41.3)	63 (46.3)	180 (42.2)
Don’t know	13 (8.5)	19 (13.8)	9 (6.6)	41 (9.6)
Missing	9 (5.9)	3 (2.2)	3 (2.2)	15 (3.5)
**Ease of compression treatment application recorded by those who self-applied treatment, *n**** (%)*	55 (35.9)	31 (22.5)	82 (60.3)	168 (39.3)
Very easy	10 (18.2)	10 (32.3)	29 (35.4)	49 (29.2)
Easy	17 (30.9)	10 (32.3)	34 (41.5)	61 (36.3)
Neutral	8 (14.5)	2 (6.5)	11 (13.4)	21 (12.5)
Difficult	15 (27.3)	2 (6.5)	6 (7.3)	23 (13.7)
Very difficult	5 (9.1)	6 (19.4)	1 (1.2)	12 (7.1)
Missing	0 (0.0)	1 (3.2)	1 (1.2)	2 (1.2)

*EBC* Evidence-based compression, consisting of 4-layer bandages or 2-layer compression hosiery, *2LB* Two-layer bandages, *CW* Compression wrap

Many trial participants had experience with several types of compression, so were able to compare CWs with other modalities. Trial participants liked that they were able to adjust the CW when it felt too tight, when it was too warm, or when it was limiting them in daily activities. Some people appreciated the ability to wear shoes and their normal clothes with the wraps, although others commented the wrap was still bulky. Additionally, the CW could be taken off for showers which participants appreciated. Most trial participants did not report issues with application of the CW, although some found it difficult initially and had to ask for support from family/friends or staff members. This positive view of CWs was reflected in questionnaire data where 60% of participants in this trial group reported applying CWs themselves, with the majority reporting they found this easy to do so (Table 2). The questionnaire responses also suggested slightly lower discomfort scores in the CWs group and lower pain scores (Table 2). This was echoed by trial participants interviewed, with those assigned to CW seeming to report less issues with comfort than those who had been allocated to EBC or 2LB.

I can’t see how you want to use anything else but the wrap to be honest. You know they are so good. I know there is plenty but they are so effective, compared to bandages anyway you know. I don’t know. I can’t see how the wraps, the convenience, the efficiency, it’s about time.—Patient 1,CW, Male

Whilst 2LH could also be removed, it was described as more difficult to apply according to both interviewed trial participants and trial questionnaire participants (Table 2).

But two-layer hosiery that I was on once it’s put on it’s absolutely fine it doesn’t move apart from, what I did find is that the top of it rolls down and digs in your leg sometimes so you do get a tourniquet effect as the top of it rolls over. Other than that, I found that really, really, good.—Patient 3 EBC, Male

Mainly because I have arthritic hands and the stockings are an absolute nightmare to get on. And if you can get them on then you can’t get them off. In fact, the second day that I managed to get it on it was so painful I had to cut it off my leg.—Patient 10 EBC, Female

Approximately one third (*n* = 3/11, 27%) of interviewed trial participants found the wraps challenging. One patient found the wrap too difficult to put on, another said their ulcer bled through the wrap into their bed. Both asked to receive different treatments as a result. Another patient said they once wore the wrap at night and woke up with the fastening from the wrap on one leg stuck to the wrap on the other leg, which could have resulted in a fall had they not noticed. Other participants mentioned that whilst they could put the wrap on, they did sometimes struggle to do this due to limited hand dexterity or hip flexibility.

Oh the wrap it was good but I found it hard to put on, even the nurses found it hard to put on, and to me it wasn’t you had to wrap it over and you had to know what wrap to I thought it was more fiddly.—Patient 4, CW, Female

Oh, I did have those [the wrap], but they didn’t work because at that time I was bleeding a lot and the blood came through everything and on my bed. So, I stopped using those and went back to the bandages.—Patient 6, CW, Female

### Staff views on compression treatments

Staff seemed more confident about applying 2LB than 4LB. Like the trial participants, staff said that bandages can be difficult for people to wear. Nurses perceived that the bulk of compression bandages prohibited people from wearing their normal shoes and/or clothes, which could be particularly problematic for those still working. Bandages were however more likely to be in stock which enabled rapid commencement of treatment. This was confirmed by the staff survey data, where most respondents noted that bandages were in stock (2LB: 67.4%(29/43) and 4LB: (52.4%, 22/42)). Additionally, nurses interviewed highlighted that bandages could be the most appropriate compression treatment at the start of treatment due to the shape of a patient’s leg (when oedema caused swelling) or due to the presence of exudate or blood. Conversely, staff reported that these clinical situations would mean that CW could not be used as they would get dirty quickly, which meant they had to be washed regularly, and as drying times could be long, patients would have to be provided with multiple wraps. Additionally, new wraps would have to be ordered once the swelling was controlled, and leg shape had changed. After a few weeks of bandages, however, people would be able to switch to 2LH or CW.

We still order those via, we order them, we still order those, but we won’t go through the GP as it takes too long. We have bandages and kits [2LH] mostly in stock, or some in stock and we get all of the treatments within a week.—Staff Site 1

I don’t understand why this man’s wound is still so wet, where it’s come …wrap but I did on Friday and it was … I couldn’t even fasten it around his leg it was too loose and that oedema was going down that worked really well but it wasn’t doing anything for the leg ulcer and he hadn’t recognised that oh I keep pulling it and try and tighten it but it keeps slipping down, he hadn’t recognised—Staff Site 5

Whilst 2LH and CW lend themselves to patient self-application, staff also recognised that they may not be suitable for everyone even if clinically appropriate, and there were differences in nursing opinions on the ease of CW application by patients; some reported application to be easy or very easy for patients, others perceived application to be difficult or very difficult for patients (Table 3). During the interviews, nurses noted for example, that not everyone would be able to apply the wrap correctly, or they may not want to see their own wounds.

And some people don’t tighten them as tight anyway do they, and I will say do them as tight as you can but their tight and my tight.—Staff Site 5

**Table 3:** Overview of outcomes staff questionnaire detailing (perceived) ease of application

	**Very easy *n* (%)**	**Easy *n* (%)**	**Neutral *n* (%)**	**Difficult *n* (%)**	**Very difficult *n* (%)**
Nurse reported ease of application**:**					
4LB	16 (43.2)	11 (29.7)	4 (10.8)	5 (13.5)	1 (2.7)
2LB	29 (67.4)	12 (27.9)	1 (2.3)	1 (2.3)	0 (0)
CW	18 (42.9)	14 (33.3)	5 (11.9)	4 (9.5)	1 (2.4)
2LH	18 (42.9)	18 (42.9)	1 (2.4)	4 (9.5)	1 (2.4)
Nurses’ perceptions of patient’s ease applying CW	2 (4.8)	16 (38.1)	10 (23.8)	11 (26.2)	3 (7.1)
Nurses’ perception of patient’s ease applying of 2LH	1 (2.4)	6 (14.6)	12 (29.3)	17 (41.5)	5 (12.2)

*4LB* Four-layer bandages, *2LB* Two-layer bandages, *CW* Compression wrap, *2LH* Two-layer hosiery

As with CWs, staff believed that 2LH was a good option overall as it allowed people to wear their clothes and shoes as normal. Similarly to CW, 2LH was also not considered to be a good compression treatment option for people whose VLUs were bleeding or where exudate was present.

Staff in FGDs reported that 2LB, 4LB and 2LH were held in stocks locally. Some sites mentioned they kept a few standard-sized CWs in stock, but others had to be ordered in. The staff survey showed some variation with 34.1% (15/44) of participants reporting availability of hosiery kits from local stocks and 55.0% (24/44) reporting a prescription being required. Some sites needed a prescription for 2LH or CWs, but they kept a small stock of 2LH or CW in stock, provided by manufacturers. These were often for standard sizes only, but if suitable for the patient, the compression treatment could immediately be applied and when the prescribed wrap arrived, it could be used to replenish stock. Prescription of treatments could however lead to delays in application of the treatment as it could take a whilst to arrive with staff mentioning that it sometimes took up to 2 weeks for treatment to arrive. One interviewed patient never received their assigned treatment.

### Compression dose

Sustained, therapeutic (strong) compression is important for healing of VLUs. The 4LB can, however, be difficult to apply correctly; one patient mentioned they noticed that staff did not seem confident when applying 4LB, and some noted bandage slippage. This was supported by the staff survey which showed more uncertainty about applying 4LB than 2LB (Table 3).

Because obviously with bandaging is on until you see the nurse next time but if it’s a wrap or hosiery you need to be quite choosy about who is going to really help themselves in a way and keep that treatment on rather than just taking it off.—Staff Site 4

Trial participants said that whilst wraps stayed in place over time, they felt looser than bandages. Additionally, trial participants said the CWs would loosen over time and the hook/loop fastening would no longer stick as well. Some trial participants struggled with applying the wrap sufficiently tightly. A further patient mentioned they were warned about tightening the wrap too much, but they did not think this could happen and said that tight was good. Staff reported that patients do not always tighten the wraps as prescribed and do not readjust the CWs during the day. They also believed patients were prone to loosen with the wrap, which could impact compression being provided, despite trial participants liking this aspect of CWs.

Staff mentioned that whilst most patients seem to wear compression treatment as intended, sometimes people came into the clinic with the CW in their hand rather than on their leg.

But there are some patients, not all, but there are some patients who for whatever reason choose to come in with the wrap in their hand as opposed to on their leg.—Staff Site 4

Most interviewed trial participants reported taking their CW off at night and reapplying in the morning. Advice provided to trial participants regarding wearing of the wrap at night seemed to differ, with some trial participants being recommended to keep it on, whilst others were not told to wear it at night. One patient initially took the wraps off at night, but that led to swelling so they decided to keep the wrap on continuously.

Yeah, the wraps are better for walking, getting in and out of the car. If the wraps are too tight when you’re doing something, I don’t know say just bending your legs to get in the car, if they’re actually too tight then you can at least undo them to get in—Patient 9, Female, 2LB

## Discussion

### Summary of results

Patient self-reported data collected via questionnaire at the VenUS 6 trial 1-month follow-up timepoint for this process evaluation showed generally high satisfaction with compression therapies evaluated in VenUS 6 but less discomfort and wound-related pain among those allocated to CW compared with the 2LH/4LB and 2LB groups. Interviewed trial participants liked that CW allowed them to wear normal shoes and clothes. In contrast to multilayer bandages trial participants could adjust CWs themselves—including loosening as well as removing completely them for showers and at night. A 2023 study involving interviews with 25 people with VLUs to explore barriers and facilitators to compression use also suggested that the ease of application of CWs was appreciated by patients [8]. However, loosening or removing CWs probably reduces the overall dose of compression received. Whilst the nurses interviewed were unsure if service users would use the CWs correctly they recognised that CWs could be a good treatment option as these may be easier to self-apply than 2LH, and potentially more comfortable.

VenUS 6 found that healing time for those assigned to the CWs was longer than for both the EBC (where participants received either 4LB or 2LH) and the 2LB [15]. On-going compression is important therapeutically in healing VLUs, and higher levels of safe compression are thought to be more effective in preventing ulcers from returning than a lower level of compression or no compression [23]. Thus, the adjustability elements of CW that trial participants find appealing to increase their comfort and potentially reduce wound-related pain could potentially impact on the total dose of compression received and be a reason that the ulcers in this arm of the trial were slower to heal.

Bandages were described as bulky and limiting people in daily activities, which has been reported previously [8]. However, once applied, staff reported that patients were less likely to change bandages themselves, waiting for a nurse to remove and reapply them. Less scope for adjustability may mean that there was a larger dose of strong compression compared with CW. However, other studies have noted patient-reported slippage of compression bandages after application, which could lead to unintended non-adherence [8].

Whilst 2LH can also be removed and applied more easily than bandages by trial participants, it supplies a predictable and consistent amount of compression and user skill does not affect this. As CWs use straps this means that there is the potential for the treatment to be looser or tighter at the wearer’s discretion or skill. However, although 2LH was considered a good treatment by both staff and trial participants because of its scope for self-application and lack of bulk, it is perceived to be difficult to apply by trial participants due to its tightness.

Returning to our logic model, the mechanisms we were primarily interested in were wearability and ease of application. We had assumed that self-application of compression wraps would be a positive finding, Although the wraps were found to be more comfortable, this was due to their adjustability however which led to a less therapeutic dose of compression and slower healing times.

### Implications for practice

We have shown that whilst there are several compression treatment options available to treat VLU, there needs to be shared decision making between health professionals and patients’ needs to consider both research evidence and a range of clinical, behavioural and preference factors when selecting the most appropriate option. Indeed, it is likely that a different treatments at different stages of healing may have to be used to provide optimal VLU treatment. The size and shape of an individual’s leg, and the amount of exudate may mean bandages with an elastic layer are the most appropriate option at early stages of treatment [7, 24]. Where non-bandage modalities are clinically possible, 2LH is known to be a cost-effective treatment, but one that can be hard for people to apply. CW are also appealing in supporting self-application but VenUS 6 and this process evaluation suggest that this seems to come with an increased risk that patients may reduce the dose of their compression by adjusting the CWs to increase comfort and are unlikely to be cost-effective compared with other options (Phung H, Soares MO, Arundel CE, Dumville JC, Saramago P, on behalf of the VenUS 6 investigators: Compression Systems for Venous Leg Ulcers: a Network Meta-Analysis and Cost-Effectiveness Analysis, unpublished).

Given current data, it is not recommended that CW be used as a first-line treatment for most people. However, for some people, this may be the only form of compression that is acceptable, and so the slower time to healing may be considered better than no compression at all. This process evaluation emphasises that when CW is used, patients need to be clearly informed about the risk associated with loosening the wraps. As noted in previous research [8], this could form part of wider approaches to educating patients about the underlying cause of their VLUs and why on-going use of compression is required to promote healing.

### Strengths and limitations

The study’s strength is the rigorous integration of trial and process evaluation data. The data show a story behind the numbers collected in the trial and provide some explanations for these. Using mixed methods for data collection allowed us to gather a wide variety of data.

Another strength is the use of focus groups for staff. The focus groups enabled staff to discuss experiences with each other, which led to more in-depth data being obtained.

A further strength of the study was that MK, who conducted most of the interviews and was not involved in the running of the trial or treatment delivery. We hope this encouraged participants to share their experiences with MK. Staff group FGDs included MK and a member of the trial coordination team based at YTU. This may have influenced the experiences shared by staff; however, as the YTU team built and sustained supportive relationships with site staff to facilitate trial conduct, we do not anticipate this influenced the experiences reported. The data collected via interviews and focus groups was analysed prior to the trial data being analysed which limited any influence of the clinical and cost effectiveness results on the process evaluation interpretations. 

One limitation includes selection bias, as we were only able to include those trial participants who consented to be approached for participation and the sampling criteria precluded us from recruiting from every participating site. Furthermore, those who consented may have been more enthusiastic about trial participation than those who did not consent, meaning the experiences of those less keen to participate in interviews may not be fully reflected.

A limitation to the study was small numbers of trial participants recruited for interviews. Those interviewed were largely representative of the overall VenUS 6 population although men were underrepresented in the interview sample (45% vs 55%) and the average age of those interviewed was slightly lower than that of the overall trial population (70.3 years vs 74.5 years). Site input came from only seven of the 33 participating sites, with limited representation of those involved in the initial pilot phase which may have limited the overall reflections on trial processes reported.

Recall bias was also a limitation. Due to the delay between trial recruitment and recruitment to the process evaluation, ulcers had often healed and participants were not able to recall trial processes or experiences. Additionally, sometimes trial participants had not used the treatment to which they were assigned. This was however mitigated as those we did recruit had often used many different types of compression due to previous ulcer treatment and/or changes in treatment. These participants were able to describe their varied experiences and which led to relatively early data saturation as no new themes emerged.

## Conclusion

Whilst patient satisfaction with CWs is very high, our trial data showed time to healing is longer than for 4LB/2LH and 2LB. Our embedded process evaluation has shown that the longer healing times for people allocated CWs may be due to users wanting to loosen up wraps to support them with everyday activities. Many CW users were told not to wear them at night, whilst both 4LB and 2LB will be worn at night.

VenUS 6 findings that CW are associated with a longer healing time for VLU and are therefore not the first-line compression treatment for most people. Compression wraps may still be an appropriate compression treatment option for some, e.g. those with limited oedema in their leg, no bleeding or exudate, and who are able and willing to change and apply the CW themselves to an adequate pressure level. It is important for nursing staff to have conversations with service users and share decisions about suitable treatment options.
